# Utilization of the out of hours service in Poland: an observational study from Krakow

**DOI:** 10.1186/1472-6963-8-212

**Published:** 2008-10-14

**Authors:** Grzegorz Margas, Adam Windak, Tomasz Tomasik

**Affiliations:** 1 Chair of Internal Medicine and Gerontology, Jagiellonian University, Medical College, Poland

## Abstract

**Background:**

In 2000 a new GP contract was introduced in Poland. It allowed GPs to subcontract out of hours care to specialized deputizing services. One such service in Kraków provides care to 61 GP practices with a population of 420 000 inhabitants. The aim of this study is to analyze seasonal and geographical variation in out of hours care use and to find the most important factors influencing it.

**Methods:**

Routinely collected data for 24 months (2003–2004) containing type, date and time of the contacts were used.

**Results:**

During the study period 238 072 contacts were recorded: 149 911 ambulatory doctor visits, 23 434 home visits and 64 727 nurse procedures. The mean rate of out of hours contacts was: for ambulatory visits 178 per 1000 inhabitants/year (varied between practices from 9 to 696), for home visits 28 (from 1 to 36) and for nurse procedures 77 (from 3 to 327). The highest rate of ambulatory visits was 739 in the age group 0–4, the lowest – 104 in the age group 45–49. The highest rate of home visits was 221 in the age group over 85. The rate of ambulatory GP visits and nurse procedures was negatively correlated with the distance between the location of GP practice and the nearest out of hours clinic. The rate of home visits was positively correlated with the age of the patient.

**Conclusion:**

Significant differences between practices suggest that non medical factors may play an important role in the patient's decision to see a GP when the surgery is closed. Their influence should be limited to make the system more efficient.

## Background

Krakow is the second biggest city in Poland with population exceeding 750 000 citizens. It is an academic city with 175 000 students and 22 institutions of high education. 34 000 temporary residents live there and majority of them are students. 66% of population is in working age, unemployment rate was 7% in 2005 and was one of the lowest in Poland. More than 99% of citizens are Polish, ethnic minorities form less than 1%.

Before the health care reform in 2000, doctors working in primary care in Poland were not responsible for out of hours care. For patients requiring medical help during nights the available resources were local hospitals and emergency ambulances. A new contract introduced night and weekend care. During night-time and weekends three different services are currently available, depending on the severity of the condition. GP out of hours service can deal with most minor conditions like infections or exacerbations of chronic diseases. However, patients are strongly advised to contact the service only for justified reasons, when home remedies or OTC treatment does not help and if there is a risk that delay in treatment may cause worsening of the disease. Information about local out of hours service is displayed outside each primary care practice. Hospital Emergency Departments („Szpitalny Oddzial Ratunkowy” – SOR) deal with more severe conditions. Their task include admissions, initial diagnostic and treatment of patients with urgent diseases, intoxication or injuries. Patient can attend the department without referral from family doctor or can be transported in the ambulance. In case of emergency conditions, like loss of consciousness, chest pain, seizures or dyspnoea ambulance service should be called.

Family doctors in Poland do not provide out of hours care themselves. As in other countries, they organize it in different ways [1-3]. In rural areas, far from other medical services, doctors work out of hours themselves, in a rota with their colleagues. Elsewhere they much more commonly contract those services with local hospitals and doctor-crewed emergency ambulance services. Another common form of out of hours care is to hire a commercial deputizing service which employs doctors and nurses to provide care.

Three major companies ("deputizing services") provide out of hours care in Krakow. Two companies contract the service with the practices located in the south of Krakow and one in the north. Location of the practice is the most important factor deciding which out of hours provider is used. No significant differences between population living in both parts of Krakow is observed. Altogether, in 2003–2004 there were six out of hours clinics in Krakow, with one or two doctors working in each of them and variable number of doctors doing home visits.

One of the deputizing services in the city of Krakow runs three ambulatory clinics situated in different places of the city, housed in large outpatient clinics. It provides out of hours service to 61 practices for a population of 420 000. All patients, whose family doctors have a contract with the service, have the right to use this service free of charge. They can use any ambulatory clinic, regardless of their address but most use their nearest.

Patients who call the service may request a home or clinic visit without prior triaging, although some visiting doctors speak to the patient before accepting the visit.

According to current regulations in Poland one doctor on duty should be responsible for a population not bigger than 20,000 inhabitants [4]. Exemption from this rule requires written permission from the National Health Fund – the exclusive public insurance company.

Out of hours ambulatory clinics are open from 6 pm to 8 am on weekdays and 24 hours a day on weekends and holidays. Two doctors and two nurses work in each clinic until 10 pm, one doctor and one nurse during the night. Doctors in the clinics work only on site, home visits are made by other doctors on call. Ambulatory clinics are run as walk-in clinics and patients do not make appointments. Patients are informed that waiting times for a home visit usually exceed 2–3 hours, while in the clinic they can be seen within one hour, except during seasonal increases in upper respiratory tract infections. In cases of emergency doctors or patients can call an ambulance [5].

In its present form this service has been functioning for seven years and is well known to patients. A characteristic of the service is unlimited access to primary care physicians: all patients are seen by a doctor on the same day they ask for a visit. It allows for exact assessment of daily demand for out of hours service and its periodical change.

The aim of this study is to answer the following questions: (1) To what extent does use of out of hours care depends on seasonal, weekly and daily variability? (2) What practice related factors influence the use of out of hours service by its patients? (3) What are the demographic characteristics of the patients using the service most frequently?

## Methods

### Data collection

In every ambulatory clinic software containing the full database of registered patients is used to record their visits. This data was used for the study. The patients were recognized by insurance number (PESEL) or by date of birth, name, surname and address. The type of a visit was recorded as follows: (i) home doctor, (ii) ambulatory clinic doctor or (iii) ambulatory nurse procedure (injection, blood pressure measurement, dressing). During registration the computer system recorded date and time of visit. In the case of doctor visit information about physician was also recorded (name and professional license number). The database of patients entitled to free of charge out of hours service was made by compilation of the lists of patients from the practices with a contract with the deputizing service. Details of patients not found in the database (emergencies and patients paying for visits) were entered by receptionists. Data collected in the years 2003 and 2004 were used in this study.

All 61 practices contracting out of hours care with the deputizing service were included in the study. The number of the patients on the list, the mean age of patients and the distance to the nearest ambulatory clinic was calculated for each practice. Distances were categorized as follows:

• category "0" – practices sharing locations with ambulatory clinics,

• categories "1", "2" and "3" – practices located respectively within the distance of 1, 2, and 3 km from the nearest ambulatory clinic,

• category "4" – practices located more than 3 km from the nearest ambulatory clinic within the borders of the city,

• category "5" – practices situated outside the city boundary.

### Statistical analysis

Analysis has been carried out separately for the three main types of visits (home doctor, ambulatory doctor, ambulatory nurse). The number of visits in each month of 2003 and 2004 was calculated as the mean daily number of visits, as the number of days differs in different months. The mean number of visits during each day of week was obtained excluding public holidays on weekdays. Visit time was shown as the time when the patient attended the ambulatory clinic or when a home visit was requested.

The percentage of population using the out of hours service in 2004 was shown by age, divided into bands of five years intervals. To assess the exact demand for out of hours care, annual visit rates per 1000 inhabitants for 2003 and 2004 were calculated. The age profile of patients visiting doctor/nurse or requesting home visit was examined and presented.

The relationship between practice characteristics and rates of visits was studied in two steps. First, correlation coefficients of these factors with number of visits per 1000 inhabitants were calculated. Afterwards, to estimate which variable best predicts rates of visits, linear regression coefficients were calculated. The statistical package SPSS 14.0 for Windows was used for data analysis.

## Results

### Types of visits

The population covered was 420 566 at the end of 2003 and 420 435 at the end of 2004 (<1% change). The total number of all out of hours visits were similar in 2003 and 2004 (Table 1). The majority of all out of hours contacts were ambulatory doctor visits. They formed 86.2% of out of hours doctor visits in 2003 and 86.8% in 2004. 83.5% of nurse procedures were injections, 9% were BP measurements and 7% were ECGs in both 2003 and 2004.

**Table 1:** Out of hours visits in years 2003–2004

	***2003***		***2004***	
Population	420 566	*Per 1000*	420 435	*Per 1000*

Ambulatory doctor	75 204	178.8	74 707	177.7
Home doctor	12 033	28.6	11 401	27.1

**Total doctor**	**87 237**	**207.4**	**86 108**	**204.8**

Nurse procedures	33 353	79.3	31 374	74.6

### Practices

The majority of practices were located within 2 km of the nearest out of hours clinic. Four practices were located in the same premises as out of hours clinics. 13.6% of practices were rural. The distribution of the population among the 61 practices included in the study and their localization are shown in Table 2.

**Table 2:** Distribution of practices: distance between practice and the nearest out of hours ambulatory

**Category**	**N = 61**	**%**	**% of population**
In the same building	4	6.6	11.4

<1 km	7	11.5	16.7

1–2 km	22	36.1	36.2

2–3 km	13	21.3	21.7

>3 km within city	7	11.5	10.1

Outside city	8	13.1	3.8

### Seasonal variations

**Figure 1:**
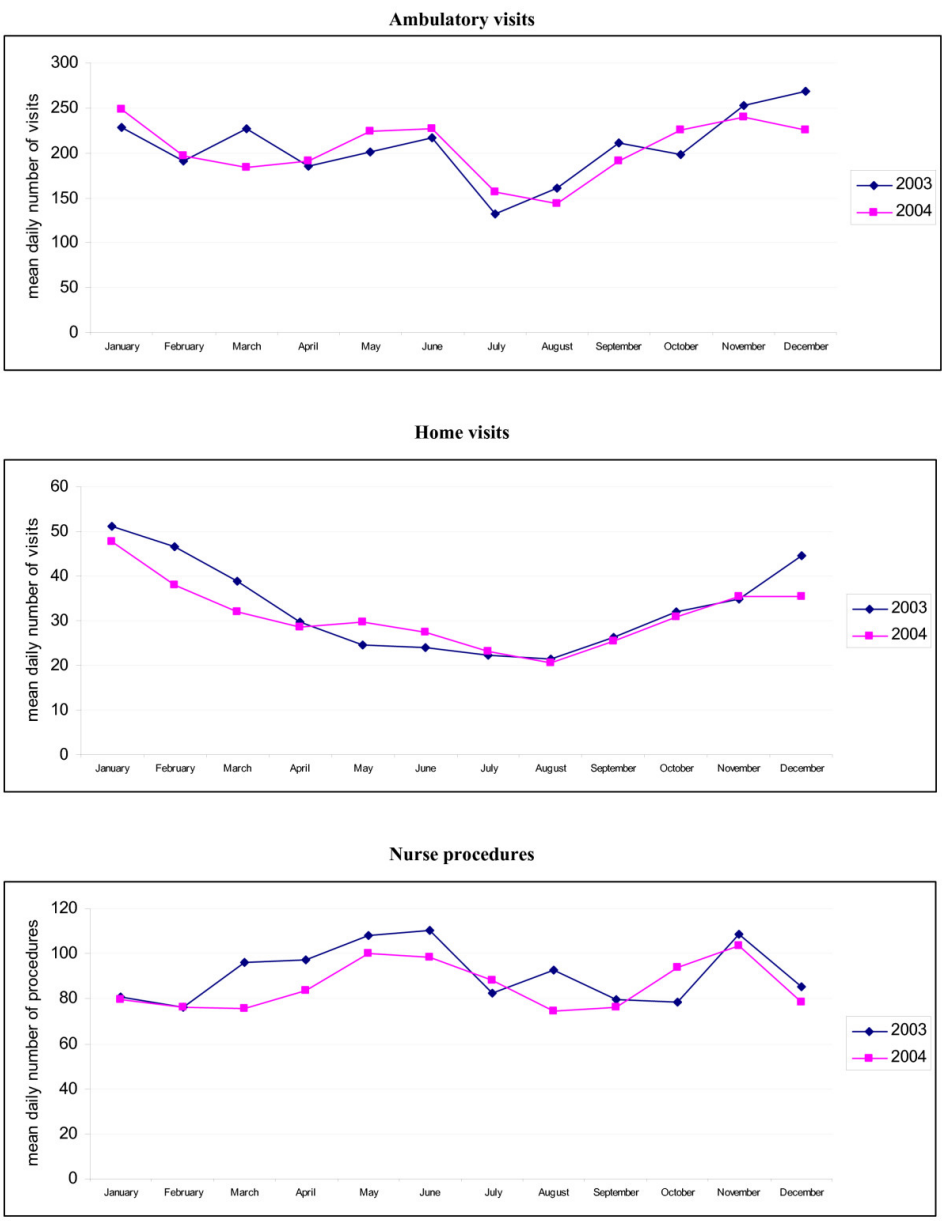
Seasonal variation in the number of visits and procedures in 2003–2004.

**Figure 2:**
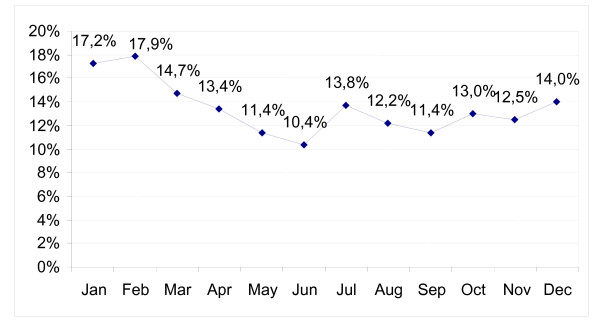
Home visits as percentage to all doctor visits (mean values from 2003–2004).

The highest daily number of ambulatory visits took place from November to January, the next highest in May and June (Figure 1). In November it was 71% higher than in the month with lowest numbers (July). The difference between months with the lowest (August) and highest (January) number of home visits was 135%. The number of performed daily ambulatory nurse procedures was highest in May and November, and lowest in February and September. The difference between the lowest and highest months was 38%. Home visits were 13.5% of all doctor encounters, highest in February (17.9%) and lowest in June (10.4%) (Figure 2).

### Daily variations

There was little variation in number of visits over the week. The highest number of daily ambulatory visits and nurse procedures took place on Friday, the highest number of home visits took place on Monday.

### Doctors and nurse workload per hour

**Figure 3:**
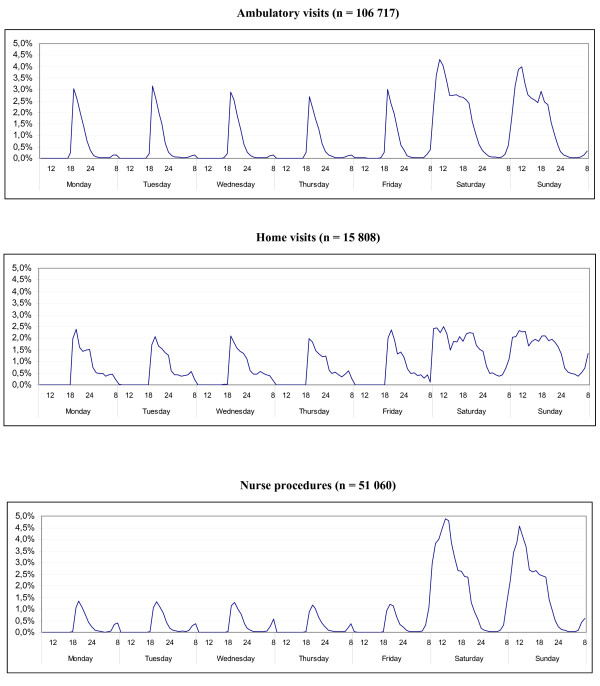
Distribution of visits over the week (public holidays on weekdays excluded).

During week days 27.7% of ambulatory visits took place within the first hour (from 6 pm to 7 pm), 79.9% within the first four hours (from 6 pm to 10 pm). 15.1% of home visits took place within the first hour, 53.4% within the first four hours. 15.6% of nurse procedures were performed within the first hour, 66.3% procedures within the first four hours. 95.1% of ambulatory visits, 78.2% of home visits and 94.6% of nurse procedures during weekends took place between 8 am and 10 pm. (Figure 3).

### Population using out of hours service

**Figure 4:**
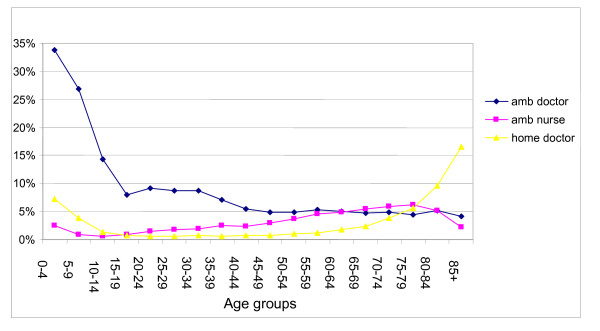
Percentage of patients in age groups using out of hours service within 12 months.

On average 12.2% of the total population entitled to the service requested a doctor's consultation out of hours in 2004 (Figure 4). 3.3% of the population requested a home visit within 12 months. Nurse procedures were made in 3.1% of population.

### Population rates of visits

**Figure 5:**
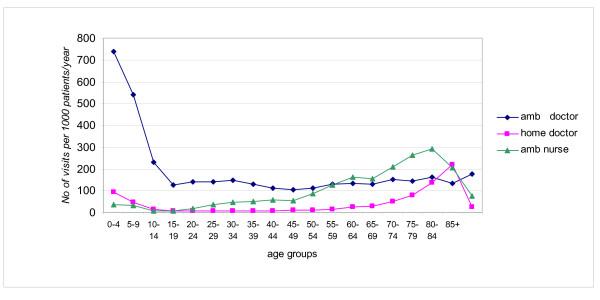
Rate of visits by age groups.

**Figure 6:**
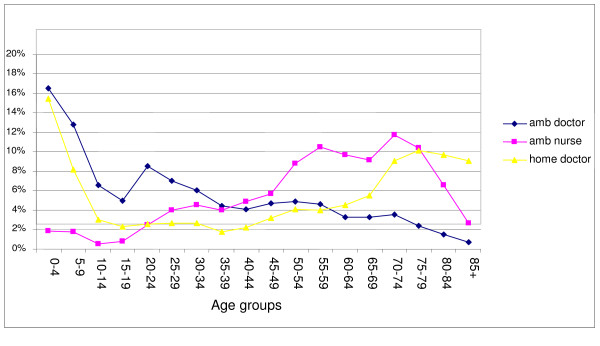
Age distribution of the population using out of hours service.

The highest rate of ambulatory visits was in the 0–4 years age group – 739 per 1000 inhabitants/year, the lowest was 104 in the 45–49 age group (Figure 5). The highest rate of doctor home visits was 221 visits per 1000/year in the group over 85 years, the lowest – 6 visits per 1000/year in the age group 20–24. The highest rate of nurse procedures was 292 in the age group 80–84, the lowest – 8 in the age group 10–19 years. The age distribution of the population using the out of hours service is shown on Figure 6.

### Ambulatory/home visits ratio

Overall, home visits were 13.5% of all doctor encounters. This number was higher in the age groups 0–4 and over 60 years, reaching 99% in the age group over 85.

### Differences between practices

**Figure 7:**
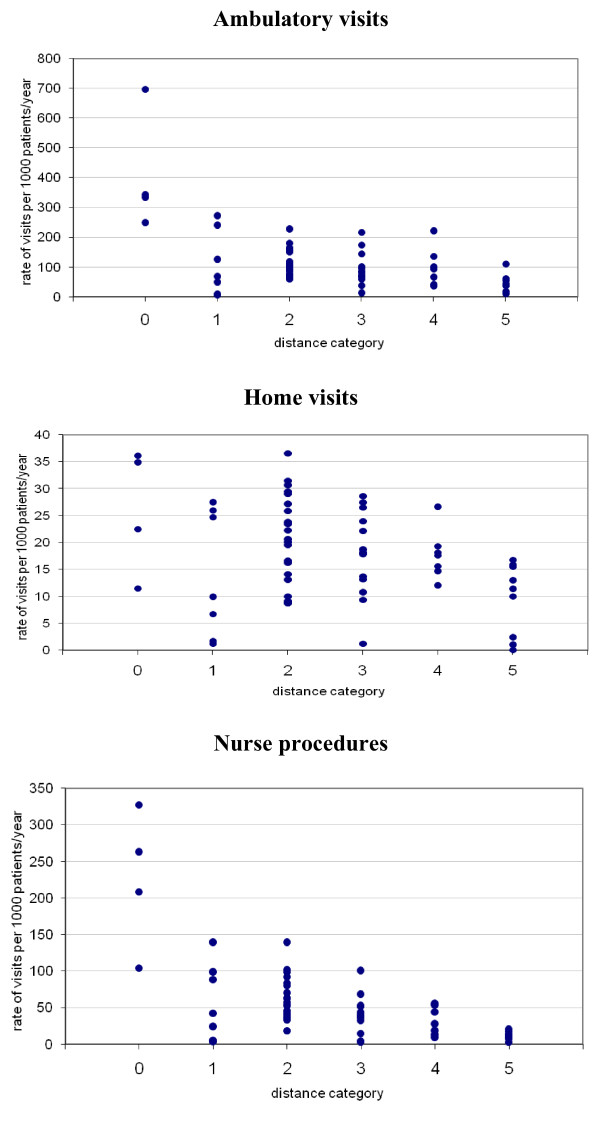
Annual rates of contacts in different distance categories of the practices.

The annual rate of ambulatory visits varied between practices from 9 to 696. The distance between patient's own practice and the nearest out of hours clinic was the most important factor influencing the annual rate of clinic visits and nurse procedures (Figure 7). This relationship was stable across age groups and was independent of the practice list size (Table 3). It was also observed after exclusion of practices located in the same premises as out-of hours clinics. The annual rate of home visits varied between practices from 1 to 36. The mean age of the patients on the list was the main determinant influencing rate of home visits. The annual rate of nurse procedures varied between practices from 3 to 327. Number of visits of patients not registered with contracting practices was below 2.5%

**Table 3:** Analysis of regression – correlation between rates of visits and practice characteristics

	**Rate of visits**					
	
	**ambulatory doctor**		**home doctor**		**Nurse procedures**	
	
	**Standardised coefficient**	p	**Standardised coefficient**	p	**Standardised coefficient**	p
List size	0.09	0.49	0.14	0.26	0.08	0.50
Mean age	-0.14	0.26	**0.50**	0.00	0.01	0.96
Distance	-**0.54**	0.00	-0.05	0.67	-**0.56**	0.00

## Discussion

Our study showed significant differences in the use of out of hours care during the year. It is in contrast with data from other countries, where little variation was found [6]. Since the differences existed during two years of observation, we can exclude the influence of incidentally increased morbidity due to a seasonal epidemic. The increased number of visits during winter months is caused by a peak of viral infections. The reason for the increased number of clinic visits during months with low incidence of respiratory tract infections (May-June) needs to be established [7].

Current requirements oblige all institutions involved in out of hours care to employ a fixed number of staff (1 doctor on duty per 20 thousands inhabitants), regardless of real needs and workload [4]. This inflexible obligation is not appropriate when demand for care changes and seasonal variations exist. Although not studied here, experience from practice shows that waiting time in the clinic may be up to three hours during winter months. In a situation when no prioritization of patients is made, it may be dangerous and be one of the causes of low satisfaction with health care services in Poland. In contrast in the UK, the time taken to triage and deal with calls is strictly monitored [8].

The annual rate of contacts in our study was low when compared to British, Dutch, Danish or Irish data [9-12]. The main reason may be that out of hours care in Poland is relatively new, introduced as a standard in the whole country in 2005. Previously out of hours care existed only in the form of hospital accident and emergency departments and ambulances staffed with doctors. It was expensive and hardly accessible. Instead, many patients used private doctors for home visits and public ambulances. We can expect growth of demand for out of hours care as observed in other countries, although data from two consecutive years did not show it [13]. As patients get more acquainted with the system, utilization should also increase.

Observed inequalities in contact rates between practices from the same area may reflect differences in their organization [14,15]. Patients having problems with accessing their own GPs during normal hours may be more likely to use the out of hours service [16,17]. It is common for patients to come to out of hours clinic before opening hours when their medical problem should be addressed by their regular doctor. The list of health issues which may be sorted in this way is limited – it is not possible to arrange a planned referral to secondary care, patients can get prescriptions and not necessarily just urgent ones [18]. Some pharmacies are open 24 hours making it possible to request a forgotten prescription for chronic disease even in the middle of the night and this happens, especially in the case of night shift workers.

In our study for calculating the distance to the nearest out of hours clinic we used indirect method. We did it for two reasons. First, calculating the exact distance for hundreds of thousands of addresses cases was not possible with available resources and software. Furthermore, registered person's address is not always a place where he or she actually lives. There are many people who live in Krakow for years, but they still keep their previous address. Calculation of the distance from a town located 50 or 250 km from Krakow could add a bias to the practices with many non-residents registered and could lead to unreliable results.

It is well known that distance to the practice is the most important factor affecting the choice of the doctor – most of the patients register within the nearest practice [19,20]. We can assume that majority of patients live within a walking distance to their practice. The distance from own practice to the nearest out of hours clinic may be a very good indicator of the distance between home to the clinic. This method has been used previously in other studies [21].

Our study showed the real demand for care, when not limited by the available number of daily appointments and when free for almost the entire population [22]. Out of hours clinics work on a drop-in basis, where no previous telephone appointment is required. Moreover, patients visiting the clinics in the middle of the night are more likely to be seen immediately instead of spending hours in the waiting room during peak times.

Our observations come from settings were there is no formal telephone triage, which is widely used e.q. in UK [23]. The reason for lack of telephone triage may be partly cultural. With one of the lowest number of telephones per 1000 of population in Europe, until the mid-nineties a telephone in Poland was still rather a luxury than a common household equipment. Most patients, in particular elderly ones, prefer face to face contact over telephone advice from a doctor or nurse [24].

Our study is based on observations from one city and may not reflect the situation in other regions of the country, especially in rural areas. However, with the introduction of new reporting software, such comparisons should be possible. Future studies should also include repeated observations in order to monitor the change in demand over time. The introduction of obligatory reporting with ICD-10 codes or other relevant classification expected in 2008 will be very valuable in explaining seasonal variations in contact rates and will provide scope for further, more comprehensive studies [25,26].

We based our study on the simple database used in the service for administrative and statistical purposes. This database contained only the basic information necessary to identify the person. Since no other information (medical history, social status etc) was stored, no other analysis was possible. However, last year a new law was introduced in Poland, which allows to store medical data in electronic form only. It should give an excellent opportunity for a new research. In our future study we plan to focus specifically on the reasons for encounters and morbidity in out of hours care.

Our study confirms the existence of the group of patients who abuse out of hours services. This group is often called "frequent attenders" [27]. To some extent their behavior may be explained by practice related factors and improving access to own doctor may help to reduce the number of unnecessary out of hours visits [15]. Raising proportion of inappropriate calls to out of hours service has been reported in previous studies [28]. Our recent study showed, that three categories of ICD-10: Z00 ("general examination and investigation of persons without complaint and reported diagnosis"), Z02 ("examination and encounter for administrative purposes") and Z29 ("need for other prophylactic measures") formed almost 10% of reasons for out of hours visits. These categories are good examples of problems which should not be addressed to the emergency service [29].

## Conclusion

Further provision of the out of hours requires some essential changes. First, information campaign for patients that out of hours service should be used only for problems, which cannot wait till own practice is open. A clear definition of such problems is necessary. Frequent reasons of encounters, like repeated prescriptions or health certificates should be excluded from this list. Patients should better understand that using out of hours care in a reasonable way is in their own interest and it can help to avoid problems with access to the GP when they really need it. For example, advice on paracetamol dosage or other antipyretics may be all that is necessary overnight for a feverish patient without other symptoms. Second, available resources should be organized with more flexible approach based on the real demand for care. Our study shows that number of calls after 10 pm decreases dramatically and most of the ambulatories can be closed at this time. To deal with late night calls less staff is required. On the other hand, staffing should be increased during seasons of increased morbidity, e.g. from upper respiratory tract infections. Out of hours performance targets should be established and waiting times should be strictly monitored. Third, implementation of telephone triage (nurse or doctor led) can limit the number of unnecessary GP visits. Observations from our study show that location of out of hours ambulatories in GP practices may lead to overuse of the service by patients from these practices. Further research is necessary to determine whether hospitals are a better place for out of hours ambulatories.

## Abbreviations

BP: blood pressure; ECG: electrocardiogram; GP: general practice, general practitioner; ICD: International Classification of Diseases; OTC: over the counter; SPSS: Statistical Package for the Social Sciences; UK: United Kingdom;

## Competing interests

The authors declare that they have no competing interests.

## Authors' contributions

GM participated in the design of the study, was responsible for data collection and analysis and drafted the manuscript. AW participated in the design of the study, in drafting and finalizing the manuscript. TT participated in drafting and finalizing the manuscript. All authors read and approved the final manuscript.

## Pre-publication history

The pre-publication history for this paper can be accessed here:


